# Predicting thresholds for population replacement gene drives

**DOI:** 10.1186/s12915-024-01823-2

**Published:** 2024-02-19

**Authors:** Anna Janzen, Ratnasri Pothula, Adam Sychla, Nathan R. Feltman, Michael J. Smanski

**Affiliations:** 1 Department of Biochemistry, Molecular Biology, and Biophysics, University of Minnesota, Minneapolis, 55455 MN USA; 2 Biotechnology Institute, University of Minnesota, Saint Paul, 55108 MN USA

**Keywords:** Population replacement, Gene drive, *Drosophila melanogaster*

## Abstract

**Background:**

Threshold-dependent gene drives (TDGDs) could be used to spread desirable traits through a population, and are likely to be less invasive and easier to control than threshold-independent gene drives. Engineered Genetic Incompatibility (EGI) is an extreme underdominance system previously demonstrated in *Drosophila melanogaster* that can function as a TDGD when EGI agents of both sexes are released into a wild-type population.

**Results:**

Here we use a single generation fitness assay to compare the fecundity, mating preferences, and temperature-dependent relative fitness to wild-type of two distinct genotypes of EGI agents. We find significant differences in the behavior/performance of these EGI agents that would not be predicted *a priori* based on their genetic design. We report a surprising temperature-dependent change in the predicted threshold for population replacement in an EGI agent that drives ectopic expression of the developmental morphogen *pyramus*.

**Conclusions:**

The single-generation fitness assay presented here could reduce the amount of time required to estimate the threshold for TDGD strategies for which hybrid genotypes are inviable. Additionally, this work underscores the importance of empirical characterization of multiple engineered lines, as behavioral differences can arise in unique genotypes for unknown reasons.

**Supplementary Information:**

The online version contains supplementary material available at 10.1186/s12915-024-01823-2.

## Background

Gene drives can direct the spread of an allele through a population, even if it carries a fitness cost that would otherwise lead to its elimination [1]. Engineered gene drives could be used to alter traits of an existing population, such as spreading a disease-refractory gene through a vector population [2, 3]. Alternatively, gene drives can be designed to suppress a population by spreading an allele that negatively impacts fitness or fertility [4]. The diverse applications of gene drives, coupled with improved tools for precision genome engineering, has driven an increase in gene drive research in the past decade.

A variety of different mechanisms have been proposed or demonstrated for engineered gene drive systems [1, 5–9]. Homing endonuclease gene drives, which spread quickly through a population by converting wild-type alleles to gene drive alleles, can be effective at low release frequencies. However, these systems will also be highly invasive and difficult to reverse. Threshold-dependent gene drives (TDGDs) are a type of replacement drive that must exceed a certain threshold frequency in a population before they will spread to fixation. They are less invasive and easier to control and reverse, and therefore are attractive alternatives. TDGDs may be ideal in many contexts because the drive is likely to be confined to the target population and can be removed by releasing wild-type organisms to reduce the gene drive to below threshold levels [10, 11]. This could be beneficial for localized and reversible population modification. Additionally, the reversibility of threshold-dependent gene drives may be advantageous from a regulatory and social perspective [12].

For TDGD systems, the threshold for population replacement is ultimately determined by the relative fitness of the biocontrol agent and wild-type populations [10, 13, 14]. There are several approaches to measuring relative fitness between two populations in laboratory settings. Laboratory measurements of relative fitness can employ (i) multi-generational mixed population experiments with continuous [15–17], semi-continuous [18], or discrete generations [17, 19–21], (ii) single-generation mixed population experiments [22], or (iii) experiments that quantify individual components that contribute to fitness (e.g., fecundity, mating competition, etc.). There are strengths and weaknesses to each of these methods, and none fully capture the aspects of fitness determinants in field settings. For example predator evasion, behavior in fluctuating and heterogeneous environments, etc. are typically not part of laboratory fitness measurements. Despite this shortcoming, laboratory measurements of relative fitness (and therefore predictions of replacement thresholds) are still useful, particularly in comparing the performance of alternative TDGD designs.

**Fig. 1:**
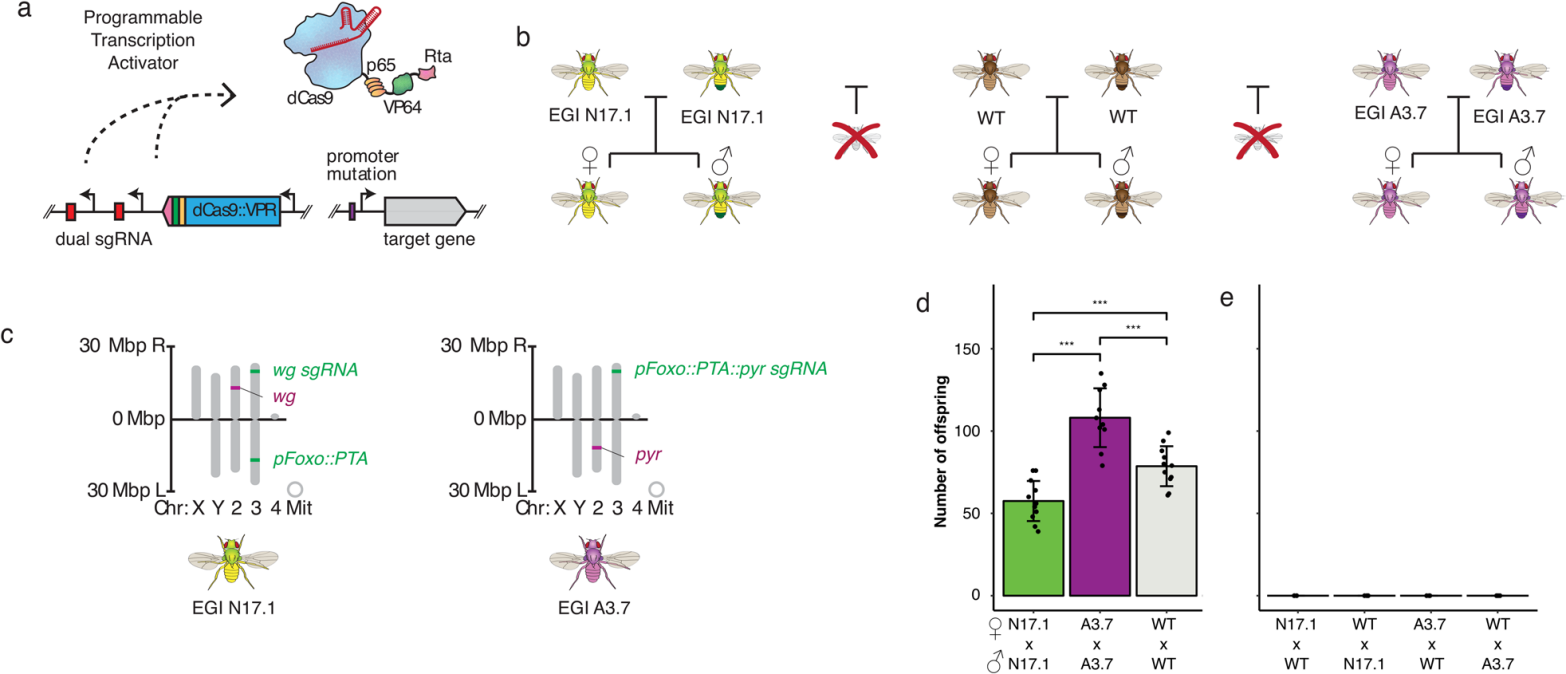
Genetics of strains involved in this study. **a** Genetic design of loci engineered for genetic incompatibility, including PTA (programmable transcription activator) expression cassette (left) and mutated promoter (right). **b** Predicted mating compatibility for strains used in this study. **c** Chromosome map of modified loci in two EGI (Engineered Genetic Incompatibility) strains used in this study, N17.1 (left) and A3.7 (right). **d** Measured fecundity of strains used in this study at 25 degrees C. Self-crosses to measure fecundity used 20 male and 20 female flies. Pairwise two-tailed Student’s t-tests were performed between each of the strains. *** = $p<0.001$. Mean and standard deviation are shown. **e** Measured fecundity of crosses between EGI and wild-type. No crosses between EGI and wild-type produced adult offspring. Crosses to measure compatibility between strains used 5 female and 3 male flies

This study is focused on determining the relative fitness of two distinct designs of Engineered Genetic Incompatibility (EGI). EGI is an extreme underdominance system that functions as a TDGD when EGI agents of both sexes are released into a wild-type population [21, 23–26]. Underdominance, also known as negative heterosis, describes true-breeding genotypes whose hybrids have a lower fitness than either parental genotype. In extreme underdominance, homozygous types (eg. EGI or wild-type) have equal fitness, and hybrids have a fitness of 0 (either due to lethality or sterility). EGI organisms are homozygous for both a haplosufficient lethal gene that is functional with only one copy and a haploinsufficient resistance allele that needs to be homozygous to provide protection. Since each EGI organism is homozygous for both alleles, they can reproduce with their like-kind with no negative impact on their fitness and fecundity. However, any matings between EGI and wild-type individuals produce inviable hybrids in which the hemizygous resistance allele cannot provide protection against the hemizygous lethal allele. Demonstrated examples of EGI utilize a dCas9-based programmable transcriptional activator (PTA) that causes lethal over or ectopic expression of endogenous genes [21, 23, 24]. This lethality is prevented in EGI individuals by homozygous mutations that prevent PTA binding to the target promoter. In theory, dCas9-based EGI could be transferred to any sexually-reproducing species.

In this study we measured two fitness components (mate preference and fecundity) using distinct genotypes of EGI and wild-type *D. melanogaster* [24]. We then performed a hybrid single-generation and discrete-multi-generation mixed population experiment to estimate the threshold of population replacement. We demonstrate that similarly engineered EGI strains differ in their fecundity and mate preference, and that this difference in fecundity can predict each strain’s threshold in laboratory experiments. We also show that our single-generation assay can reproducibly show differences in the thresholds of similarly-engineered EGI lines.

## Results

### Fecundity

To determine the fecundity of the EGI lines N17.1 and A3.7 [24] (for a description of their genotypes see Methods), we set up vials with 20 male and 20 female virgin flies from one strain and counted the number of offspring produced per vial. 10-12 replicates were performed for each strain. N17.1 vials produced an average of 57.5 offspring (SD=12.2). This is significantly less than the wild-type (Oregon-R) vials ($p=0.00044$, Student’s t-test), which had 78.6 offspring on average (SD=12.1). A3.7 had significantly more offspring than Oregon-R, with 108.1 offspring on average (SD=17.8) ($p=0.00027$, t-test) (Fig. 1d). To confirm that hybrids between wild-type and EGI are not viable, we also mated 5 EGI females with 3 *w*^1118^ males and quantified the number of offspring produced per vial. Fewer flies were used for the compatibility tests than the fecundity test due to limited numbers of virgin females available. No crosses between EGI and *w*^1118^ produced adult offspring (Fig. 1d). However, subsequent experiments in which we explored the impact of changing environments (namely temperature) on TDGD performance metrics revealed adult hybrid offspring between EGI and wild-type flies at certain temperatures (Supplementary Fig. 2).

**Fig. 2:**
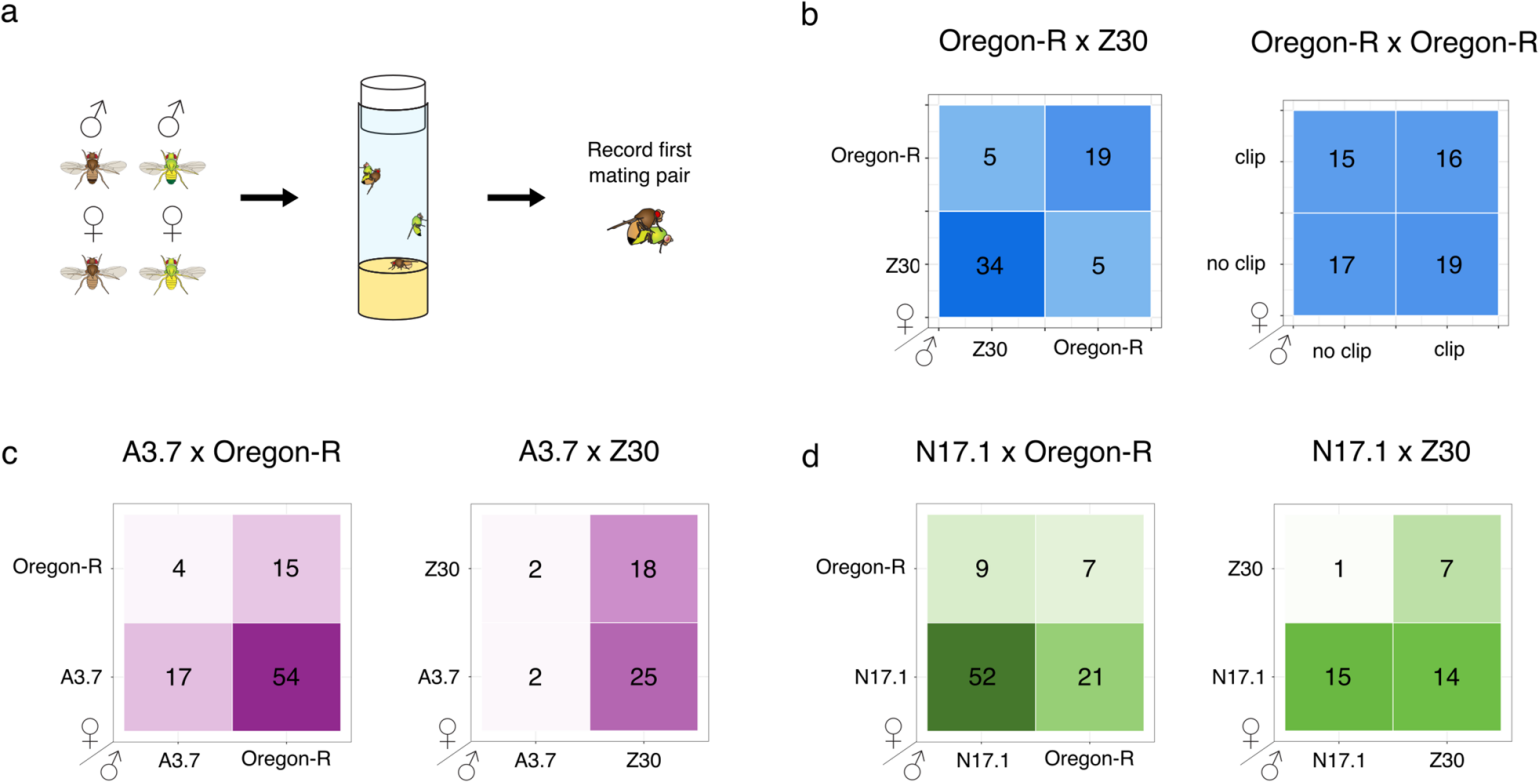
Mate preference between engineered and two types of wild-type flies. **a** Experimental design for mate preference assay. **b** Data for positive (Oregon-R x Z30, left) and negative (Oregon-R x Oregon-R, right) control mate preference assays. Heatmaps show the number of mating events observed for each possible pair. **c** Mate preference results from A3.7 with Oregon-R ($n = 90$) and Z30 ($n = 47$). **d** Mate preference results from N17.1 and Oregon-R ($n = 89$) and N17.1 and Z30 ($n = 37$)

### Mate preference

Before determining the mate preference of EGI flies, we confirmed our ability to detect non-random mating using the Z30 fly strain, which has previously been found to mate assortatively [27]. To measure the mate preference of the Z30 flies, we set up vials with one Oregon-R male, one Z30 male, one Oregon-R female, and one Z30 female and recorded the first pair to mate (Fig. 2a). This four-fly test has previously been used to measure mating preferences in *D. melanogaster* [28–30]. The first pair to mate was a Z30 male and a Z30 female 53.9% of the time, which is consistent with the previously described assortative mating phenotype. The mate preference observed was found to significantly differ from random mating (chi-squared = 36.5, df = 3, $p<$ 10^-5^). The mating results support assortative mating of these two genotypes ($p = 0.047$, Fisher’s exact test) (Fig. 2b, left).

To tell the two fly lines apart during this experiment, we used surgical clippers to remove the distal portion of the wing for flies from one of the two lines. To ensure that this wing clip was not impacting the mating behavior of the flies and to serve as a negative control, we repeated the mate choice experiment using all Oregon-R flies, half of which had clipped wings. There was no significant deviation from random mating (chi-squared = 0.52, df = 3, $p = 0.93$) (Fig. 2b, right). Despite this, we alternated the strain with wing clips in each replicate to control for any potential impact.

Next we repeated the mate choice experiment with an Oregon-R male, EGI male, Oregon-R female, and EGI female in each vial. EGI flies were engineered using the *w*^1118^ line as a starting point. This fly line has a loss of function mutation in the *white* gene that has previously been found to influence male courtship behavior [31, 32]. However, all EGI lines in this paper have the mini-*white* gene on recombinant constructs for strain construction/screening purposes. This has previously been found to rescue defects in copulation success caused by the mutation in *white* [32]. Therefore, we expected males and females from both EGI lines to mate randomly with Oregon-R flies. However, we found that both EGI lines (A3.7 and N17.1) exhibited a unique mating phenotype. A3.7 female flies showed a strong early mating preference over wild-type females, being the first to mate 71 out of 90 times. Oregon-R males similarly showed a strong early mating preference over A3.7 males, mating first 69 out of 90 times. Overall, 60.0% ($n=54$) of matings were between female A3.7 and male Oregon-R flies, 4.4% ($n=4$) of matings were between an Oregon-R female and A3.7 male, 18.8% ($n=17$) of mating were between two A3.7 flies, and 16.6% ($n=15$) of matings were between two Oregon-R flies (Fig. 2c, left). These are strongly non-random (chi-squared = 63.2, df = 3, $p < 10^{-5}$), but do not show evidence for assortative mating ($p = 0.99$, Fisher’s exact test).

**Fig. 3:**
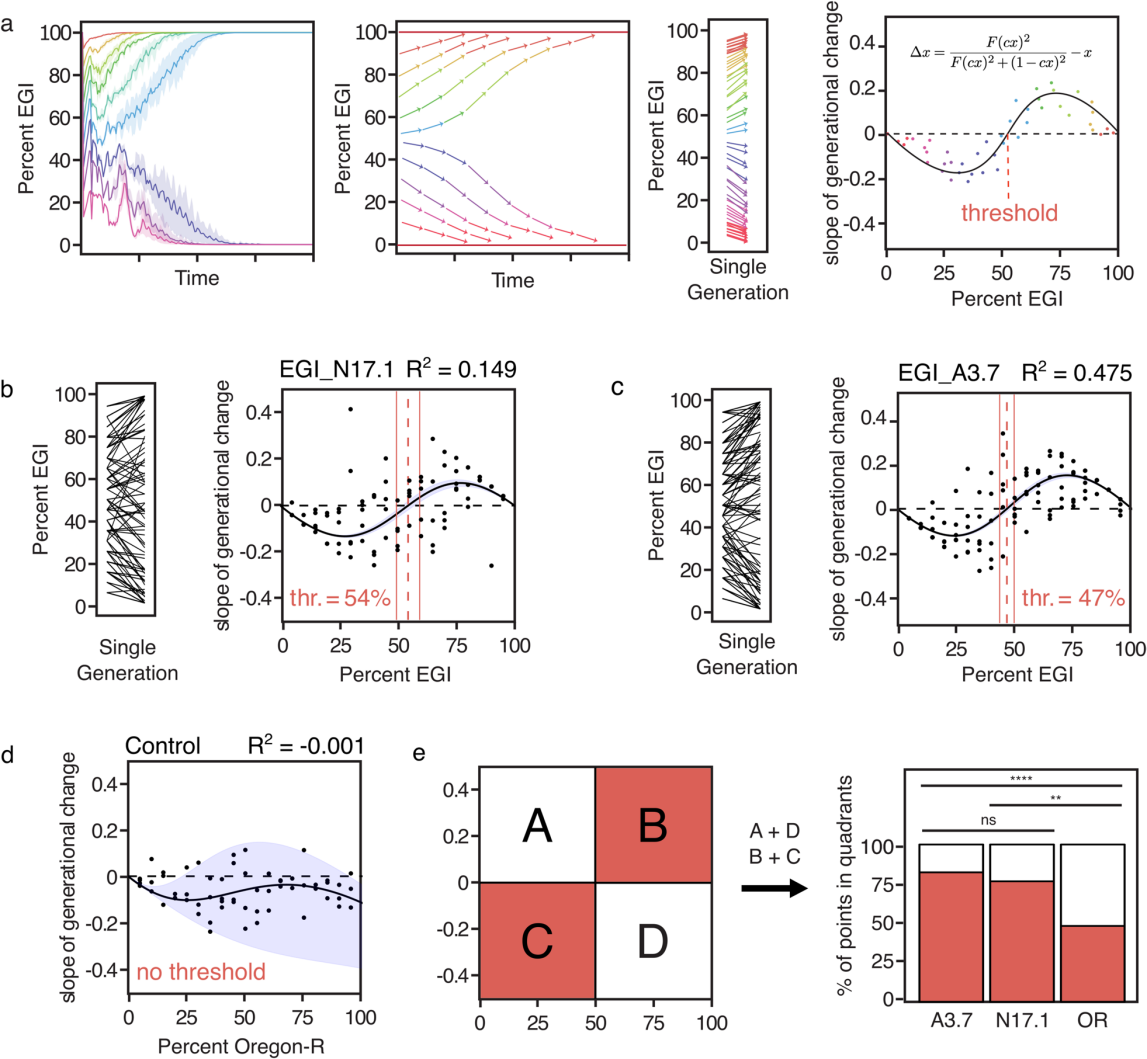
Threshold-dependent gene drive measurements. **a** Method of determining replacement threshold in single-generation experiment. Left: Agent-based model simulation of threshold-dependent gene drive activity for an underdominance-based drive. Left-center: Bi-stable behavior of multi-generation mixed population with underdominant genotypes can be captured by measuring changes in population frequency in each generation (arrows). Right-center: Measurements of threshold made for this study involved 19 different starting frequencies of EGI:wild-type, for which the single-generation change in frequency was recorded. Right: Plots of the rate-of-change of population frequency as a function of starting population frequency are fitted with an equation. Experimental estimation of population replacement threshold for EGI A3.7 (**b**) and N17.1 (**c**). Dotted lines indicate the predicted threshold, solid lines indicate Standard Error Mean (SEM). Light blue indicates 68% confidence interval (i.e., plust or minus one standard deviation). **d** Rate of change of Oregon-R as a function of starting population frequency in control experiment. **e** Left: Example of quadrants used to analyze differences in experimental and control data. Right: Percent of points in quadrants A+D and B+C for A3.7, N17.1, and control (Oregon-R) experiments. ns = no significance, ** = $p<0.01$, **** = $p<0.0001$

N17.1 flies similarly exhibited strong non-random mating behavior (chi-squared = 58.2, df = 3, $p < 10^{-5}$). We observed 58.4% ($n=52$) of all mating events between male and female N17.1 flies, 23.6% ($n=21$) between a N17.1 female and Oregon-R male, 10.1% ($n=9$) between an Oregon-R female and N17.1 male, and 7.9% ($n=7$) between two Oregon-R flies. The high frequency of N17.1 self-mating represents a slight but significant assortative mating phenotype ($p = 0.025$, Fisher’s exact test) (Fig. 2d, left).

We also performed this assay using EGI and Z30 flies to determine how the presence of a confirmed assortative mating phenotype in wild-type flies would impact the mate choice of EGI flies. We expected that we would see assortative mating behavior in these experiments. For A3.7 (Fig. 2c, right), there was a similar wild-type male preference for early mating over the A3.7 male (43 out of 47), but no appreciable preference for females. Overall, 53.2% ($n=25$) of matings were between male Z30 and female A3.7, 4.3% ($n=2$) between male A3.7 and female Z30, 4.3% ($n=2$) between two A3.7 flies, and 38.3% ($n=18$) between two Z30 flies (Fig. 2c, right). This is strongly non-random (chi-squared = 34.5, df = 3, $p < 10^{-5}$), but does not reflect assortative mating ($p = 0.99$, Fisher’s exact test).

For N17.1 and Z30 (Fig. 2d, right), 18.9% ($n=7$) of matings were between two Z30 flies, 37.8% ($n=14$) were between an N17.1 female and Z30 male, 2.7% ($n=1$) were between a Z30 female and N17.1 male, and 40.5% ($n=15$) were between two N17.1 flies. Once again, this shows non-random mating behavior (chi-squared = 13.9, df = 3, $p < 10^{-2}$), but not assortative mating ($p = 0.10$, Fisher’s exact test).

### Determining the threshold

Empirical determination of gene drive thresholds typically requires establishing multiple populations of gene drive and wild-type individuals at varying ratios, then following the population over multiple generations. This type of experiment can be performed as a mixed-generation cage trial [16, 17] in which a population is continuously maintained until one genotype goes to fixation, or as a discrete-generation trial [17, 19–21] in which the offspring from one generation are removed from the population, counted to determine the ratio of gene drive to wild-type individuals, and used to start the next population at the same ratio. Mixed-generation cage trials are generally considered to give a more accurate representation of the threshold of a gene drive because they account for differences in longevity, eclosion rate, and lifetime fecundity between strains. In comparison, discrete-generation experiments are logistically simpler but less representative of field performance. In both types of experiment, determining the threshold is time-consuming. Each generation of flies takes about 2 weeks to develop to adulthood, so it can take multiple months before one genotype goes to fixation and the threshold can be determined.

**Fig. 4:**
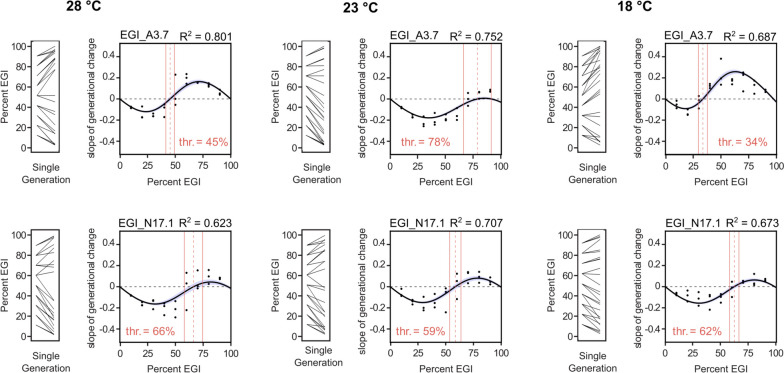
Threshold-dependent gene drive measurements at different temperatures. Measurements of threshold made for this experiment involved 10 different starting frequencies of EGI:wild-type, for which the single-generation change in frequency was recorded. Experimental measurement of population replacement threshold for EGI A3.7 (top) and N17.1 (bottom) at 28, 23 and 18^∘^C were shown along with R-square (R^2^) values and threshold (thr.)

To accelerate the process of determining the threshold, we developed a new assay as an alternative to the discrete-generation experiment. In this assay, we empirically determined how the ratio of EGI to Oregon-R flies changes over one generation at 9 different initial frequencies. We then used those data to estimate the threshold for A3.7 and N17.1 by fitting the data to an equation (derivation detailed in Methods).

We started the experiment with populations of 20 male and 20 female virgin flies in ratios of EGI:wild-type between 2:38 (i.e. 5% EGI) and 38:2 (i.e., 95% EGI), at steps of 5% population frequency at 25°C. The flies were allowed to mate and lay eggs for 5 days, then all adults were removed from the vials. On day 15, the number of Oregon-R and EGI progeny in each vial were counted. The change of the percent of EGI flies in the population was plotted as a function of the initial ratio of EGI (Fig. 3b-d).

An equation based on Mendelian inheritance patterns was fit through this plot (Fig. 3). This approach allows data from each of the measured population frequencies to factor equally in the calculation of the drive threshold. The R^2^ of the model fit was 0.149 for the N17.1 data and 0.475 for the A3.7 data. The place where the function crosses the line y = 0 is considered the threshold. This is the point at which the per-generation change in population frequency changes from a negative to a positive number. The thresholds for N17.1 and A3.7 were determined to be 54 ± 4.9% and 47 ± 3.1% respectively.

As a negative control, we repeated the 40-fly experiment using two wild-type fly lines, Oregon-R and *w*^1118^ (Fig. 3d). To compare these results to the experimental data, we counted the number of plotted points in four quadrants of the coordinate plane. The quadrants were determined by dividing the plot horizontally at 0 and vertically at the threshold for the 2 experimental plots and 0.5 for the control plot. Threshold-dependent gene drives are expected to have a negative fitness below the threshold and a positive fitness above the threshold. In other words, we would expect the majority of data points to be in quadrants B and C (Fig. 3e, left). Therefore, we used the ratio of points in quadrants B and C to those in A and D to compare the results of the experimental and control data. The data collected from A3.7 and N17.1 had a significantly higher proportion of points in quadrants B and C when compared to the control (A3.7: $p = 0.00003$, N17.1: $p = 0.0015$, pairwise z-test of two proportions)(Fig. 3e).

To try to improve reproducibilty of the data, we increased the number of flies to 60 male and 60 female virgin flies per replicate in ratios of EGI:wild-type between 12:108 (i.e. 10% EGI) and 108:12 (i.e. 90% EGI) with increments of 10% population frequency. Experiments were carried out in bottles instead of vials to accommodate the larger number of flies.

Next, we wanted to see whether this method could reproducibly detect changes in threshold of EGI lines due to changes in temperature. We conducted three replicates of the the 120-fly experiment at 18^∘^C, 23^∘^C and 28^∘^C. The thresholds were calculated as explained above.

The thresholds for A3.7 varied greatly with temperature. The thresholds for A3.7 at 28^∘^C, 23^∘^C and 18^∘^C were estimated to be 45 ± 4%, 78 ± 12% and 34 ± 4% respectively (Fig. 4 top panel). On the other hand, temperature did not impact the threshold of N17.1. The thresholds for N17.1 at 28^∘^C, 23^∘^C and 18^∘^C were determined to be 66 ± 8%, 59 ± 5% and 62 ± 4% respectively (Fig. 4 bottom panel).

When compared to the 40-fly experiment, the 120-fly experiment tended to give more reproducible results for each initial ratio, and the R^2^ values were much higher. However, the confidence intervals for the threshold prediction were not any tighter for the 120-fly experiment. This is likely due to the large number of replicates (6-7 per starting ratio) performed for the 40-fly experiment, compared to 3 for the larger experiment, suggesting that only a few replicates of the 120-fly experiment gives results as precise as twice as many replicates of the 40-fly experiment.

**Fig. 5:**
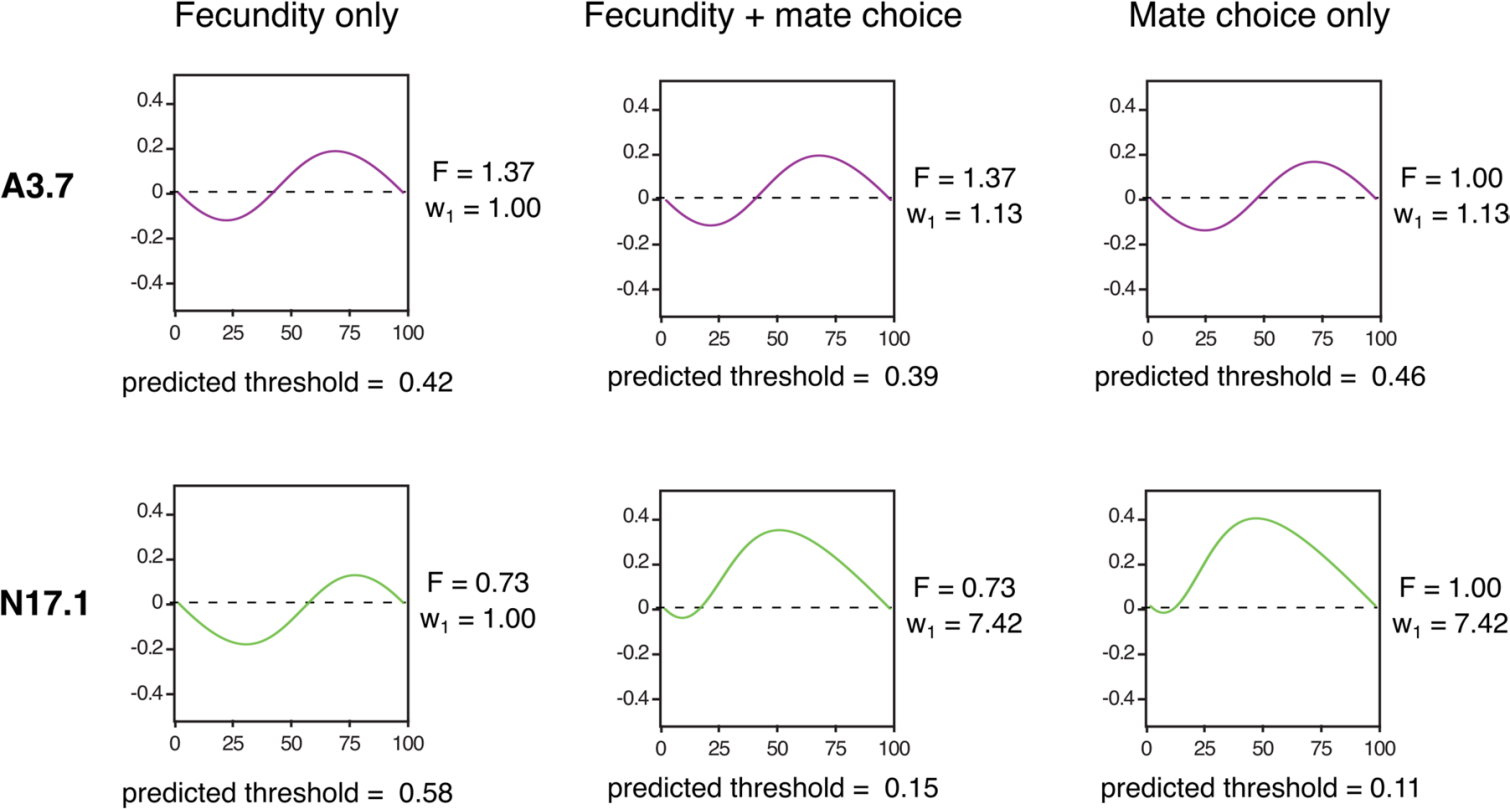
Threshold for A3.7 and N17.1 predicted using model. Left: fecundity data only, Center: mate choice and fecundity data, Right: mate choice data only. Equation 2 was used for fecundity only prediction, Eq. 3 was used for fecundity and mate choice and mate choice only predictions (See Supplementary Note 1)

### Gene drive model

Lastly, we retrospectively tested whether the isolated measurements of fecundity and mate preference could be used to predict gene drive thresholds as measured using our single-generation assay. We developed a mathematical model with relative fecundity and mate preference as its only free parameters (Eq. 11).

First, we used this equation to estimate the threshold using only fecundity data by setting the relative preference for assortative mating ($w_1$) to 1. Using the measured fecundity data from each strain at 25^∘^C (Fig. 1d), the model predicted the threshold for A3.7 to be 42%. For N17.1, the threshold was predicted to be 58% (Fig. 5, left).

To determine whether incorporating mate choice data would increase the accuracy of our model, we calculated the predicted threshold using the calculated *F* and $w_1$ values. We found that this new predicted threshold was farther from the measured threshold and therefore less accurate than the one generated using only fecundity data. Using this equation, the model predicted the threshold for A3.7 to be 39% and the threshold for N17.1 to be 15% (Fig. 5, center). We also estimated the threshold using only mate preference data. To do this, we set the fecundity of both EGI lines as equal to wild-type (F = 1). Using this data, the predicted threshold was 46% for A3.7 and 11% for N17.1 (Fig. 5, right).

## Discussion

The threshold of population replacement for a TDGD is largely determined by the relative fitness of the biocontrol agents and wild-type. Many factors influence the relative fitness, including mating competitiveness, non-random mating, fecundity, longevity, and survival rates [33–36]. Predictions of relative fitness, and therefore the threshold for TDGD replacement can be made based off of laboratory measurements of these factors, or by mixed population experiments that measure the change in population frequency across generations.

Mixed population studies used to measure relative fitness include single-generation [22] and multi-generation experiments. Multi-generation studies can further be classified as discrete, semi-continuous, or continuous based on how or if individuals from different generations are allowed to interact. In discrete-generation experiments, there is no mating between flies from different generations. The change in population frequency of each genotype is determined from a single generation experiment, and then that frequency is used to seed the next generation [19]. In semi-continuous experiments, offspring from one generation are mixed into cages or bottles of future generations at predetermined ratios [18]. In continuous experiments, populations are maintained in small or large cages in which individuals are able to interbreed across generations to an extent that is limited only by the organism’s biology [15, 16]. Semi-continuous and continuous generation measurements account for fitness components such as longevity and length of fertile period that are missed in single-generation or discrete-generation measurements. Conversely, single-generation experiments are less resource intensive and can be replicated in greater numbers to better measure variability.

The approach that we describe and demonstrate in this study lies somewhere between a traditional single-generation fitness measurement [22] and a discrete- multigeneration measurement. Instead of starting only with a 50:50 mixture of TDGD and wild-type individuals, as was done by Jungen and Hartl, we test nineteen different starting population frequencies from 5% EGI to 95% EGI. This allows the experiment to account for behaviors such as the minority-male preference [37]. In discrete multi-generation approaches, similar experiments are performed eventually, they are just spread out over a longer time since each new population frequency is set up sequentially. Our method is suitable for gene drives such as extreme underdominant drives in which heterozygotes are not viable, but would not work for drives in which heterozygotes are viable and can impact the frequency trajectory of future generations.

Compared to semi-continuous or continuous multigenerational approaches, our method does not measure as many components of fitness (e.g., longevity and length of female fertility). However, according to Hartl and Jungen, single-generation tests like this do include the major components of fitness [18]. One benefit of the quicker assays is that they are less affected by evolutionary forces, which have been observed to change relative fitness of tested strains partway through multigenerational experiments [18]. Additionally, our single-generation approach is substantially less sensitive to fungal/bacterial contamination, which has decreased the number of replicates reported for previous discrete multi-generational studies [21]. Because we did not determine the threshold for EGI flies and wild-type by any other means, we cannot say whether these predictions of threshold frequency would agree or disagree with values obtained from multigenerational tests.

One drawback to both the rate-of-change approach to measure gene drive threshold (demonstrated here) and the discrete, multi-generation approaches demonstrated previously is that they fail to account for the impact on total population numbers throughout a replacement campaign. At relative ratios near the replacement threshold, a substantial degree of population suppression is expected, as a result of inviable hybrid offspring. This, in theory, would affect the relative impact of immigration from outside the control area. The consequences of such an impact should be explored through future modeling studies.

Assortative mating in the wild-type population, whether due to an existing allele or evolved in response to release of a gene drive [38], can also cause an EGI-based TDGD to fail by reducing the chance that wild-type individuals mate with EGI individuals. Assortative mating in wild-type populations has previously been noted during sterile insect technique (SIT) programs, including in the melon fly (*Bactrocera cucurbitae*) [39] and medfly (*Ceratitis capitata*) [40].

We observed dramatic non-random mating in a conventional dual-choice mate selection assay. However, because the measured fecundity did a better job of predicting the relative fitness of EGI and wild-type lines than did fecundity-plus-mate choice, we are suspect of the assortative mating results. For A3.7, all 3 model predictions gave predictions similar to the estimated threshold of 47%. For N17.1, we found that the most accurate prediction was obtained when using fecundity data only. Incorporating the mate preference data resulted in predictions of very low thresholds that were not corroborated with empirical results. It is noteworthy that this assay is highly dependent on the kinetics of mating and may not be relevant in larger populations where being first to mate is less important.

Mating behavior is known to impact the success of insect control strategies [33, 34]. However, mate choice can be difficult to accurately measure and quantify [30, 41]. Given the discordance between the measured mate preferences, and the measured replacement thresholds, particularly for N17.1, we believe that the 4-fly mate preference assay used here is a poor predictor of mating behavior in cage or field settings. Only the first pair to mate was recorded in the 4-fly experiment, so the observed mating patterns may have reflected propensity or speed of mating more than mate preference [42]. In the gene drive experiment, the adult flies were given 5 days to mate, so speed of mating may have been less relevant under these conditions. Additionally, female *D. melanogaster* often mate with multiple males and can store sperm from multiple matings, with paternity of the female’s offspring biased in favor of the last male. Therefore, only measuring the first mating may not be the most accurate way to predict the genotypes of offspring because it does not consider remating. As the measured strength of mating preferences is dependent on experimental design, using a variety of different tests (eg. 4-fly test, 3-fly test for male and female mate choice, and no-choice tests) could help to obtain a more accurate picture of the true mate preferences of the lines being tested [30]. Using accurate measurements of mate preference will be important for future attempts to model the effectiveness of biocontrol strategies. In future work, emerging technologies that allow for computer vision-based tracking of flies over long time intervals could be used to provide more relevant measurements of individual behaviors [43, 44].

This work highlights the importance of characterizing multiple independently engineered insect strains, even if they are engineered in similar ways and are not expected to be phenotypically different. In this study, we found that two similarly engineered EGI lines exhibit different mate preferences and fecundity. There was no reason to predict this *a priori* based on the distinct targets for engineered overexpression. The distinct performances highlights the importance to empirically characterize multiple lines when developing biocontrol agents, even if the designs seem equivalent. Our results suggest that a difference in fecundity is responsible for the different thresholds of these two lines. A3.7, the EGI line with higher fecundity than Oregon-R, had a lower replacement threshold compared to N17.1, showing that phenotypic differences measured in lab experiments can cause differences in the performance of a gene drive. This finding has also been noted by another group who saw varying effectiveness of EGI lines, with the estimated thresholds of their lines ranging from 61% to 90% [21].

We sought to test the performance of EGI lines at different temperatures in part because of the reported temperature-dependent activity of Cas9 [45, 46]. We were surprised to observe that similarly engineered EGI strains showed differences in the way their predicted replacement threshold changed as a function of temperature. Our results suggest that the lower threshold of A3.7 at 18^∘^C was due to significantly higher fecundity of A3.7 than Oregon-R at that temperature. Similarly, A3.7 had a 45% threshold at 28^∘^C, at which both A3.7 and Oregon-R had similar fecundity measures (Fig. 4 and Supplemental Fig. 1). However, even though A3.7 had significantly higher fecundity compared to Oregon-R at 23^∘^C (Supplemental Fig. 1), the threshold of A3.7 was still very high. This can be explained by the observation that the total number of A3.7 adult offspring emerging from EGI-dominated proportions (e.g., starting frequencies of 60-90% EGI) were always lower compared to total number of Oregon-R adult offspring emerged from wild-type-dominated proportions (e.g., starting frequencies 10-50% EGI) (Supplementary Data). This single threshold-prediction experiment is parsimonious with the observed non-random mating measured for A3.7 (Fig. 2c), but it is strange that the thresholds determined at other temperatures do not seem to be impacted by the same non-random mating. It is known that mate selection in *Drosophila* is impacted by cuticular hydrocarbon (CHC) composition [47, 48], which in turn might be altered by changing environmental conditions such as temperature, humidity or day length [49–53]. On the other hand, temperature did not influence the threshold of N17.1 as much.

While we do not think the mating preference results measured in the 4-fly test are particularly relevant to field-release scenarios, the unexpected and stark differences between similarly engineered strains is still noteworthy. N17.1 showed an assortative mating phenotype, whereas A3.7 females tended to prefer mating with wild-type males over A3.7 males. The reasons for these unique preferences are not immediately obvious. Mate selection in *Drosophila* species is known to be influenced by many factors, including cuticular hydrocarbon (CHC) composition [47, 48], social factors like mate choice copying [54, 55], and diet/microbiome (although this has proven difficult to replicate) [28, 29, 56, 57]. Additionally, a number of genetic loci that impact courtship and mate choice have been identified in *Drosophila* species [58, 59]. Although both EGI lines were originally generated from the same laboratory strain (*w*^1118^), it is possible that due to a founder effect, the two lines could have different alleles at a locus involved in mate choice, causing the difference in mate preference and fecundity that was observed. Alternatively, the location of the dCas9-VPR construct or off-target gRNA bindng could be affecting the expression of genes involved in these traits.

In summary, here we demonstrate a method to estimate the threshold of threshold-dependent gene drive using a single-generation assay. We show that the empirical results for threshold differ from those predicted using behavior measurements at the individual-level. Additionally, we show that similarly engineered EGI lines display distinct properties and behaviors that would not have been predicted *a priori*, including changes in threshold at different temperatures.

## Conclusions

The threshold for population replacement can be estimated in a single generation by fitting a curve to data showing the rate of change of population frequency as a function of starting frequency of a population replacement biocontrol agent. Engineered Genetic Incompatibility can drive population replacement with thresholds of approximately 50%, but this threshold is temperature dependent in one EGI line. Differences in fecundity, mate preference, and estimated population replacement threshold in distinct lines of genetically engineered biocontrol agents underscores the importance of empirical characterization of several different genotypes to uncover properties that would not be predicted *a priori*.

## Methods

### Genotypes of flies used in this study

Five fly lines were used in this study. Oregon-R is a cosmopolitan wild-type that was collected from Roseburg, OR in 1925 and is commonly used in research settings [60]. Z30 was isolated from the Sengwa Wildlife Reserve in Zimbabwe, Africa in 1990 and is known to show assortative mating behavior with Oregon R [27]. N17.1 and A3.7 are two EGI lines with distinct genetic designs [24] (Fig. 1a-c). Finally, *w*^1118^ was used as the wild-type for the 25°C incompatibility assay. It has a partial deletion of the *w* locus on the X chromosome.

N17.1 has two engineered loci on chromosome 3: a dCas-VPR programmable transcriptional activator (PTA) driven by the *FoxO* promoter on the left arm and a pair of sgRNAs with spacer sequences complementary to the *wingless* (*wg*) promoter. The sgRNAs are driven by distinct U6 promoters on the right arm. The *wg* target gene is on chromosome 2.

A3.7 uses a similar PTA design to ectopically express the *pyramus* (*pyr*) gene. Unlike N17.1, both the PTA and sgRNAs are expressed from the same locus on the right arm of chromosome 3. We have previously reported the behavior of these fly lines and their mutual incompatibility [24].

### Fly rearing and media

Fly strains were maintained at 25^∘^C with 12 hour light/dark cycles. All experiments were conducted using 3-10 day post eclosion virgin flies in vials with Nutri-Fly Bloomington Formula (Genesee Scientific, 66-121) food with 0.1% Tegosept (Genesee Scientific, 20-258) and 0.05 M propionic acid (Sigma, 402907). Oregon-R flies were used as wild-type unless noted otherwise.

### Mate preference

Before beginning the experiment, the males and females from one strain had the tips of their wings clipped off to allow for identification. Wing clipping has not been found to significantly impact courtship or mating success [61]. The strain with clipped wings was alternated for each replicate. First, one female from each strain was loaded into a vial with food and dry yeast, followed by one male from each strain. The wing clipping pattern of the first pair to mate in each vial was recorded and used to identify the mating pair. Pairs were given 3 hours to mate; any vials that did not mate during this time were discarded. This experiment was conducted at room temperature. To determine whether the mating pattern observed differed significantly from random mating, we used a chi-squared test. To test for assortative mating, we used Fisher’s exact test.

### Fecundity

The fecundity of A3.7, N17.1, and Oregon-R flies was measured by mating 20 male and 20 female flies from the same strain. The flies were allowed to mate in vials for 5 days, then the adults were removed. On day 15 the number of adult offspring was counted. A3.7, N17.1, and Oregon-R had 10, 12, and 11 replicates performed respectively.

To measure the effect of temperature on the fecundity of A3.7, N17.1, and Oregon-R flies, 60 virgin females were allowed to mate with 60 virgin males of the same strain for 5 days in a bottle. The adults were removed after 5 days and the emerged progeny were counted. The experiment was set up in triplicates at 18^∘^C, 23^∘^C, 25^∘^C, and 28^∘^C for each strain. Data were analysed using Two-way ANOVA and multiple comparisons were made using Tukey’s test.

### Mating compatibility

To confirm that the EGI lines used were incompatible with wild-type, A3.7 and N17.1 males and virgin females were crossed to wild-type. Five virgin females and 3 males were added to a vial, then removed after 5 days. On day 15 the number of adult offspring was counted.

To ensure the incompatibility of EGI A3.7 and N17.1 with Oregon-R under different temperatures, 60 males, or 60 virgin females of EGI lines were crossed to 60 virgin females or 60 males of Oregon-R, respectively. The males and females were transferred to bottles and allowed to mate and lay eggs for 5 days, after which the adults were removed, and the progeny were counted as they emerged. The experiment was conducted in triplicates at 18^∘^C, 23^∘^C, 25^∘^C, and 28^∘^C for each strain.

### Threshold dependent gene drive

Populations of 20 male and 20 female virgin flies were set up in ratios between 5:95 and 95:5 EGI:wild-type. The adults were allowed to mate and lay eggs for 5 days. On day 15, the adult offspring were removed and frozen so that the percent of EGI flies in the population could be determined later. When determining the number of EGI and Oregon-R flies in the second generation, A3.7 flies were differentiated from wild-type flies by eye color and N17.1 flies were identified by their expression of RFP. The data from this one-generation experiment was used in a mathematical model to estimate the threshold for each EGI line.

To determine the effect of temperature on the threshold, 60 EGI strain and 60 Oregon-R virgin flies were set up between 10:90 EGI:wild-type and 90:10 EGI:wild-type proportions per bottle in triplicates at 18^∘^C, 23^∘^C, and 28^∘^C. The adults were allowed to mate and lay eggs for 5 days and then they were removed, and the adult progeny was collected and frozen as they emerged. The emerged male and female EGI and Oregon-R flies were counted.

As a negative control to confirm that this method can detect when there is no gene drive in a population, this experiment was repeated using 2 wild-type strains: Oregon-R, which has red eyes, and *w*^1118^, which has white eyes due to a mutation in the *white* gene on the X chromosome. The number of male progeny with red eyes or white eyes was counted and used in the model. After plotting the change in the percent EGI in the population between generations, the number of points in each quadrant of the coordinate plane was counted. On the y-axis quadrants were divided at the 50% EGI mark, and on the x-axis they were divided at 0. A population with a gene drive would be expected to have more points in quadrants 1 and 3, whereas a population without a gene drive would be expected to have approximately an equal number of points in each quadrant. A pairwise z-test of two proportions was used to compare the proportion of points in each group of quadrants between EGI and wild-type lines.

### Modeling for threshold dependent gene drive experiments

We used a mathematical model fit to the empirical TDGD data (above) to determine the threshold for each EGI line tested. At first, we assumed random mate selection but allowed for differences in likelihood to mate and fecundity between EGI and wild-type organisms. We used two free parameters: *F*, the measured fecundity of EGI individuals relative to Oregon-R, and *c*, which modified the population frequency term for EGI flies and can be considered a propensity for an EGI fly to mate (similar to models of other underdominance drive systems) [62]. Note that the fecundity of wild-type has been arbitrarily set to 1, so it is not apparent in the equations below. With *x* as the frequency of EGI agents in the population, the adjusted probabilities of each mating event occurring are given by:

1 $$\begin{aligned} p\text {(EGI x EGI)} = (cx)^2 \end{aligned}$$

2 $$\begin{aligned} p\text {(wt x wt)} = (1-cx)^2 \end{aligned}$$

3 $$\begin{aligned} p\text {(EGI x wt)} = 2(cx)(1-cx) \end{aligned}$$

Since the survivorship of hybrid flies resulting from EGI x wild-type crosses is 0, the frequency of EGI in the next generation ($x_{t+1}$) is given by:

4 $$\begin{aligned} x_{t+1} = \frac{F(cx)^2}{F(cx)^2 + (1-cx)^2} \end{aligned}$$

Subtracting the original ratio of EGI individuals in the population gives the change in the frequency of EGI individuals between generations. The threshold is the unstable equilibrium where this equation equals 0:

5 $$\begin{aligned} \Delta x = \frac{F(cx)^2}{F(cx)^2+(1-cx)^2}-x \end{aligned}$$

Above this threshold, EGI individuals are more likely to spread to fixation, and below this threshold EGI individuals are more likely to be eliminated from the population. In order to determine the threshold of the EGI flies, we used the Gauss-Newton algorithm to fit Eq. 5 to data from the threshold dependent gene drive experiment showing the percent change in the frequency of EGI flies after one generation. Confidence intervals were plotted at one standard deviation above and below the fit parameter value(s) (e.g. in the case of the two parameter model the upper threshold was plotted at $F+\sigma _F$ and $c+\sigma _c$). Upon determining the parameters of best fit, $R^2$ was determined as [63]:

$$\begin{aligned} R^2 = 1 - \frac{\Sigma (y_i-f_i)^2}{\Sigma (y_i-\overline{y})^2} \end{aligned}$$

The threshold was then calculated by finding zeros to the equation. This approach allowed for the fit of functions in Fig. 3 to account for general differences in fecundity or mating preference, but does not take into account the empirically measured differences in mate preference (see below).

Next, we replaced the general term for mating likelihood, *c*, with terms $w_1$, $w_2$, and $w_3$ that account for the specific mating frequency of each male x female combination from a two-genotype population. Specifically, we weighted each possible mating relative to the wild-type by wild-type cross.

6 $$\begin{aligned} z = w_1 x^2+(w_2+w_3)(x(1-x))+(1-x)^2 \end{aligned}$$

7 $$\begin{aligned} p\text {(EGI x EGI)} = \frac{w_1 x^2}{z} \end{aligned}$$

8 $$\begin{aligned} p\text {(EGI x wt)} = \frac{w_2 x(1-x)}{z} \end{aligned}$$

9 $$\begin{aligned} p\text {(wt x EGI)} = \frac{w_3 x(1-x)}{z} \end{aligned}$$

10 $$\begin{aligned} p\text {(wt x wt)} = \frac{(1-x)^2}{z} \end{aligned}$$

Here, $w_n$ represents the relative preference for that mating pair as compared to the wild-type by wild-type cross. Using these adjusted mating probabilities and again accounting for the fact that hybrids do not survive, the model equation including mate choice and fecundity parameters is:

11 $$\begin{aligned} \Delta x = \frac{F*\frac{w_1 x^2}{z}}{F*\frac{w_1 x^2}{z}+\frac{(1-x)^2}{z}} - x \end{aligned}$$

This simplifies to:

12 $$\begin{aligned} \Delta x= \frac{F w_1 x^2}{F w_1 x^2+(1-x)^2} - x \end{aligned}$$

As Eq. 12 shows, only the mate preference of EGI x EGI ($w_1$) relative to wild-type x wild-type impacts the threshold for drive replacement. Data from the mate preference and gene drive experiments were used with this equation to estimate the threshold.

### Statistical analysis

Several different methods were used throughout this study to determine statistical significance. For comparison of numerical results for which multiple biological replicate experiments produce data that is normally distributed, we perform a pairwise Student’s t-test (e.g., Fig. 1d). We performed two statistical analyses for each mate preference experiment (e.g., Fig. 2). First, we performed a Chi-squared analysis to determine if the observed mate selection differed from random sampling (i.e., 25% frequency in each of the four possible categories). We empirically determined p-values in Python by simulating 100,000 experiments with the same number of replicates (*N*) as the physical experiment. This produced a minimum p-value that could be attained of 10^-5^. To determine whether assortative mating occurred, we used the Fischer’s exact test on the 2x2 contingency tables shown in Fig. 2. To determine whether the observed mate preference patterns differed from random mating, we used a Chi-squared test on the same contingency tables. Lastly, a z-test of two proportions was used to compare the fraction of data points in respective quadrants in Fig. 3e.

### Supplementary information

**Additional file 1.**

**Additional file 2.**

## Data Availability

All data in this study is available within the paper and Supplementary Information. R and Python codes are available at github.com/smanskiLab/Threshold_Dependent_Gene_Drive. EGI lines A3.7 ($\#$93598) and N17.1 ($\#$93596) are available at the Bloomington Drosophila Stock Center. Transgenic sequences are available in a previous publication describing the creation of these strains [24]. All supplementary information is found in two supplementary files. The file titled “Additional File 1. pdf” includes Supplementary Fig. S1 - Fecundity of strains at different temperatures and Supplementary Fig. S2 - Fecundity of crosses between EGI and wild-type at different temperatures. The file titled “Additional File 2. xlsx” contains all raw data.

## Abbreviations

TDGD: Threshold-dependent gene drive
EGI: Engineered genetic incompatibility
PTA: Programmable transcriptional activator
SD: Standard deviation
