# Supplementary material for: Predicting thresholds for population replacement gene drives
Source: BMC Biol. 2024 Feb 19;22:40. doi: 10.1186/s12915-024-01823-2 (PMC10875781; doi:10.1186/s12915-024-01823-2)
Supplement: Supplementary file 1 — Additional file 1. [file 12915_2024_1823_MOESM1_ESM.pdf]

Supplementary Note 1: Supplementary Figure 1

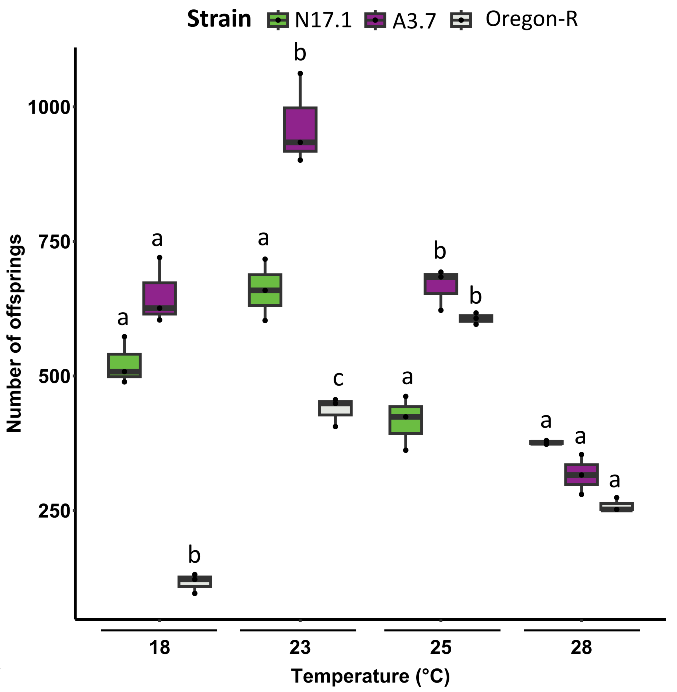

Supplemental Figure 1. Fecundity of Strains at Different Temperatures Measured fecundity of strains used in this study at 18, 23, 25 and 28 ℃. Fecundity was measured by Self crossing 60 males and 60 females of each strain. Lower and upper box boundaries represent 25th and 75th percentiles, respectively, line inside box indicate the median, lower and upper error lines represent 10th and 90th percentiles, respectively. Letters indicate significant difference among strains with in each temperature (*i.e.,* at 18 ℃ the two groups marked with 'a' are not significantly different, but the group marked 'b' is) (Two-way ANOVA, n = 3, p < 0.05, Tukey's test).

Supplementary Note 2: Supplementary Figure 2

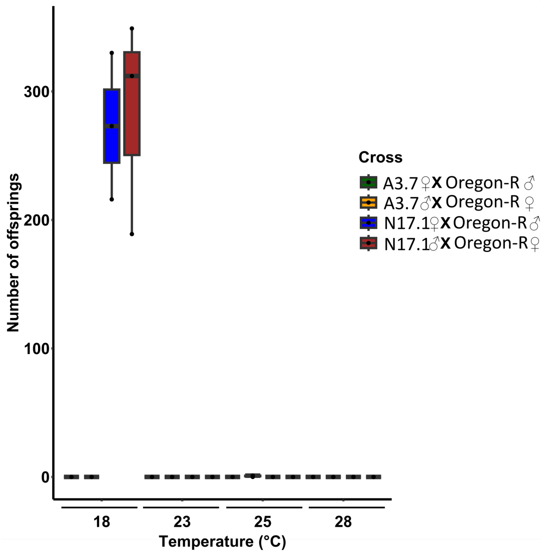

Supplemental Figure 2. Fecundity of Crosses between EGI and Wild-type at Different Temperatures Crosses to measure compatibility were made using 60 males and 60 female flies at 18, 23, 25 and 28 ℃. Crosses between N17.1 and wild-type yielded adult offspring at 18 ℃ and crosses between A3.7 male and wild-type female had adult offspring at 25 ℃. Other crosses had no adult offspring.
